# *Macrosiphoniella remaudierei*, a new species of aphid on *Helichrysum* in Iran (Hemiptera, Aphididae)

**DOI:** 10.3897/zookeys.640.9329

**Published:** 2016-12-13

**Authors:** Sebastiano Barbagallo, Juan M. Nieto Nafría

**Affiliations:** 1 Dipartimento di Agricoltura, Alimentazione e Ambiente, University of Catania, Italy; 2 Departamento de Biodiversidad y Gestión Ambiental, University of León, Spain

**Keywords:** Hemiptera, Sternorrhyncha, new records, Aphididae, Iran

## Abstract

A new species of aphid, belonging to the genus *Macrosiphoniella* Del Guercio, 1911, is described using three samples collected in Iran on *Helichrysum armenium* (Asteraceae, Inuleae) by the late Prof. G. Remaudière. Both apterous and alate viviparous females of the new taxon, *Macrosiphoniella remaudierei* **sp. n.**, are described and compared to corresponding morphs of the closely allied *Macrosiphoniella aetnensis* and to other congeneric aphid species on *Helichrysum* in the Palaearctic region. Type specimens are now stored in the Muséum national d’Histoire naturelle in Paris.

## Introduction

The large aphid genus *Macrosiphoniella* Del Guercio, 1911 (Hemiptera Aphididae) currently contains at least eight species from *Helichrysum* (Asteraceae, Inuleae), distributed over much of the old world, ranging from N Europe southwards to Macaronesia and eastwards to Central Asia and West Siberia. See Blackman and Eastop (2006-2015) for a general key to aphid species on *Helichrysum*. Four species belong to the subgenus Ramitrichophorus Hille Ris Lambers, 1947, and the remaining four (currently attributed to *Macrosiphoniella* nominotypical subgenus) probably represent two distinct genetic lineages. One lineage includes *Macrosiphoniella helichrysi* Remaudière, 1952, and *Macrosiphoniella madeirensis* Aguiar & Ilharco, 2005, which are both brown in colour and very similar to each other. The other lineage contains *Macrosiphoniella aetnensis* Barbagallo, 1968, which is green in life and is apparently limited to southern Europe (Italy, France and Spain), to which the taxon described here as a new species is added. Specimens of the new taxon were collected in Iran and are indeed very similar in morphology to those of *Macrosiphoniella aetnensis*; they were borrowed from the collection of the late Prof. G. Remaudière, which is stored at the Muséum national d’Histoire naturelle (Paris). The relevant species were detected by the second author together with a few hand-written notes by Prof. G. Remaudière, in which he pointed out the main differences, mostly focussing on the divergent aspects of body pigmentation and appendages from *Macrosiphoniella aetnensis*. Prof. Remaudière provisionally labelled the Iranian aphid as a new subspecies of *Macrosiphoniella aetnensis*. Further examination of morphological features and collection of biometric data from the available specimens of the new taxon, however, suggest that it is a full species rather than a subspecies. Furthermore, the two compared taxa, as far as is known, have separate distributions (SW Europe for *Macrosiphoniella aetnensis* and Iran for *Macrosiphoniella remaudierei*, respectively) following their host species *Helichrysum* (i.e. *Helichrysum italicum* and allied taxa for *Macrosiphoniella aetnensis* and *Helichrysum armenium* for *Macrosiphoniella remaudierei*). The range of distribution of the two host plants do not overlap, which would allow different pathways of differentiation for the two strictly allied aphid taxa.

## Materials and methods

Available material of the new taxon consists of 53 adult specimens from three samples collected in Iran (for details see Types paragraph). All of the specimens having been mounted on slides. The specimens are mainly apterous except for two alate viviparous females and a few small nymphs. Type specimens of the very similar *Macrosiphoniella aetnensis* (see Barbagallo 1968) were compared, including a large sample (containing more than 60 specimens, viviparous apterae and alatae) borrowed from the Muséum national d’Histoire naturelle (Paris). The latter specimens were collected from *Helichrysum* sp. (Le-Grau-du-Roi, Gard, France, 13.IV.1970) by Prof. G. Remaudière. Detailed biometric data were collected using an optical microscope from 20 randomly selected specimens for each of the two aphid taxa and then evaluated. The remaining available specimens of both taxa, though not individually measured, were also morphologically examined. Morphological features were compared to further species borrowed from specimens stored in the authors’ collection and/or to published morphological descriptions whenever needed.

## Results

A conclusive evaluation of the morpho-biometric data for the species allows the description of the following new aphid taxon.

### Macrosiphoniella remaudierei sp. n.

Taxon classificationAnimaliaHemipteraAphididae

http://zoobank.org/CCEF664C-3A26-47B8-9689-F52E3EC0A32D

**Figure 1.**
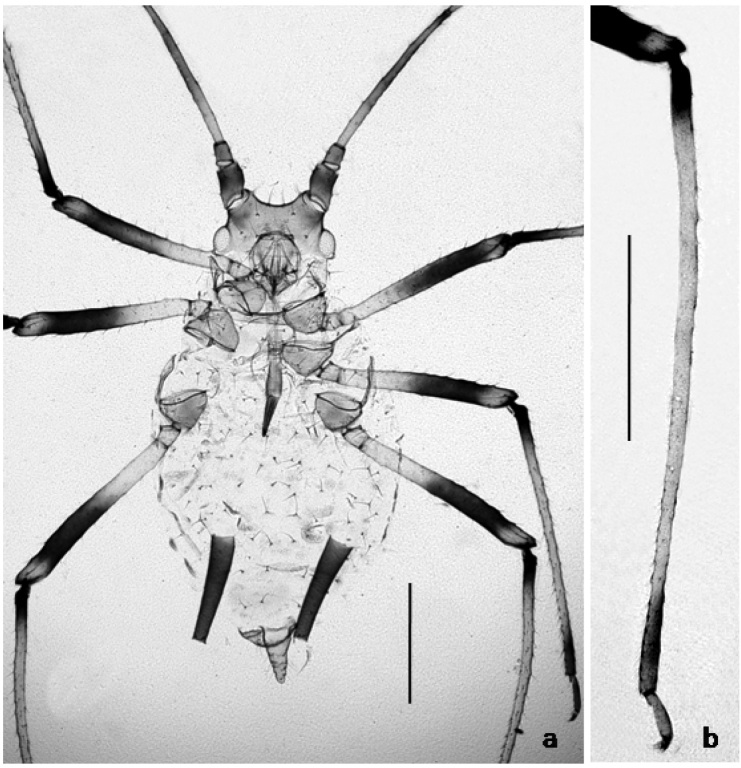
**a** *Macrosiphoniella remaudierei* sp. n., apterous viviparous: body shape and pigmentation **b** hind tibia. Scale bars 0.5 mm.

Figures 1 , 3 ; Table 1

#### Diagnosis.

Body pale in apterae morph with slightly brownish head in macerated specimens; dorsal body setae without pigmented spots at their basis; antesiphunculur sclerites lacking. Legs with coxae slightly pigmented; trochanters pale; tibiae with a pigmented basal part less extensive in length than their brown distal part. Subgenital plate pale. Cauda much less pigmented than siphunculi; their ratio (cauda length/siphuncular length) 0.7 or less. Antennal joint III with (12)18–37 secondary sensoria, extended over 66–93% of its basal length. Alate morph (two specimens seen) with 36–40 secondary sensoria on III antennal joint; otherwise similar to apterae. This new species resembles very much *Macrosiphoniella aetnensis* from which it can be separated as quoted in the taxonomy paragraph.

#### Description.

***Apterous viviparous female*** (51 specimens). Macerated body length: 1.65–2.25 mm. Colour in life unknown, but probably green with a waxy secretion, as in the very similar *Macrosiphoniella aetnensis*. Head pigmented brown, darker on anterior half and with a deep frontal sinus (0.22–0.35 of the distance between the inner apices of antennal tubercles), median frontal tubercle lacking. Thorax and abdomen pale, lacking any dorsal pigmentation, including the pre-siphuncular sclerites (although the area is quite sclerified) or the scleroids at the base of the dorsal setae. Very small, wart-like tubercles at the base of the setae irregularly present from the metathorax to the 7^th^ urotergite. Dorsal body setae are blunt or faintly flattened at apex, mainly on frons and along the spinal area of the thorax and abdomen, yet becoming gradually more acute on marginal areas of the body and terminal segments of the abdomen. Their maximum lengths are: 62–82 µm (1.80–2.70 of the basal articular diameter of antennal joint III) on the frons, 53–86 µm on the 2^nd^ and 3^rd^ urotergites, and 60–86 µm on the 8^th^ urotergite. The latter urite bears 5–7 (usually 6) quite curved setae.

*Antennae* 2.37–3.18 mm long, or 1.27–1.57 of body length, are mostly pigmented, except for a more or less extended paler basal part of joints III, IV, and occasionally V. Antennal joint III (0.56–0.76 mm), 0.77–0.98 times the processus terminalis of joint VI (0.69–1.00 mm); the latter is 3.90–5.00 times the length of the basal part (0.16–0.20 mm) of the same joint. Secondary sensoria on joint III only, ranging from 12–37 in number (most frequently 22–30), but only rarely less than 20, and then in smaller specimens, down to 1.65–1.80 mm of the body length. These sensoria are flat or very slightly protruding and of variable size, up to 0.35–0.66 of the basal diameter of joint III; they are distributed on the ventral side of the joint along 0.66–0.95 of its length. Primary rhinaria are regularly ciliated. Antennal setae are quite stout, blunt, or subcapitate at the apex, particularly on the first two joints, then gradually more or less flattened and more acute towards the antennal apex. Those on joint III have a maximum length of 26–40 µm, or 0.80–1.28 of the basal articular diameter of the same joint.

The *rostrum* extends to behind the posterior margin of the hind coxae and is well pigmented towards its distal part. The ultimate rostral joint is stiletto-shaped, 0.145–0.195 mm long, or 1.10–1.36 of the second joint of hind tarsus (including the unguiferous); it usually bears 6 supplementary hairs (rarely 5 or 7), of which 4 are anterior and 1–3 are dorsal and smaller.

*Legs* with coxae moderately pigmented (more or less similar to head capsule); trochanters are usually pale. Femora are also pale at their one-third basal part and well pigmented (darker than the coxae) towards their distal part, where a depigmented macula is more or less evident on all legs. Dorsal femoral hairs are more or less blunt or subcapitate, and the ventral hairs are always more acute at the apex; the former have a maximum length of 32–50 µm, 0.54–0.85 of the trochantro-femoral suture. Tibiae are unpigmented for most of their length, except for the brown basal and distal parts. The basal pigmentation is usually less extensive than the distal brown part and no longer than about 2.0–2.5 of the tibial width at the same basal point on the hind legs. Tibial setae are mostly pointed except for the more proximal on the outer side, which are quite blunt or subcapitate at the apex. Longest tibial hairs reach 34–50 µm, 0.84–1.20 of the median tibial diameter on the hind legs. Tarsi are brown, with their second joint (0.130–0.154 mm) 0.72–0.90 of the basal part of antennal joint VI. First tarsal chaetotaxy is 3:3:3, which is usual for the genus.

*Siphunculi* (0.34–0.50 mm) are well pigmented, 0.15–0.23 of body length, 3.19–4.30 of their basal diameter, and slightly tapering towards the apex, which is flangeless. They are usually straight, but sometimes slightly curved outwards; reticulated distal area 0.36–0.53 of their total length.

*Cauda* (0.18–0.30 mm) is pale or very slightly pigmented, digitiform in shape, and faintly constricted, or almost tongue-shaped and not constricted (mostly in smaller specimens); it is 0.53–0.68 of the siphunculi and bears 9–14 (most frequently 10–12) long and quite curved setae.

The *genital plate* is pale and faintly sclerified (although not pigmented), bearing 2–5 discal (2 long and 0–3 much shorter) and 8–12 marginal setae.

***Alate viviparous female*** (from 2 specimens). Colour in life unknown. Head and thorax are pigmented brown; abdomen is pale as in apterae, except for the fairly brownish genital plate. Papillae on abdominal dorsum are not evident. Antennae 2.79–3.43 mm and 1.36–1.44 of body length, darker than in apterae with quite pale basal parts of joints III and IV; joint III has 36–40 secondary sensoria distributed along its length. All veins of the forewings have a narrow brown border, mostly evident on Cu1 and Cu2. Pigmented legs are very similar to apterae, with darker coxae and subdistal parts of the femora. Siphunculi are more slender than in apterae and cover 0.17–0.18 of the body length. Cauda, 0.66–0.70 of the siphunculi, is darker than in apterae but paler than the siphunculi.

All other morphological features are very similar to those of the apterous viviparous female. Body length: 2.05–2.38 mm.

**Figure 2.**
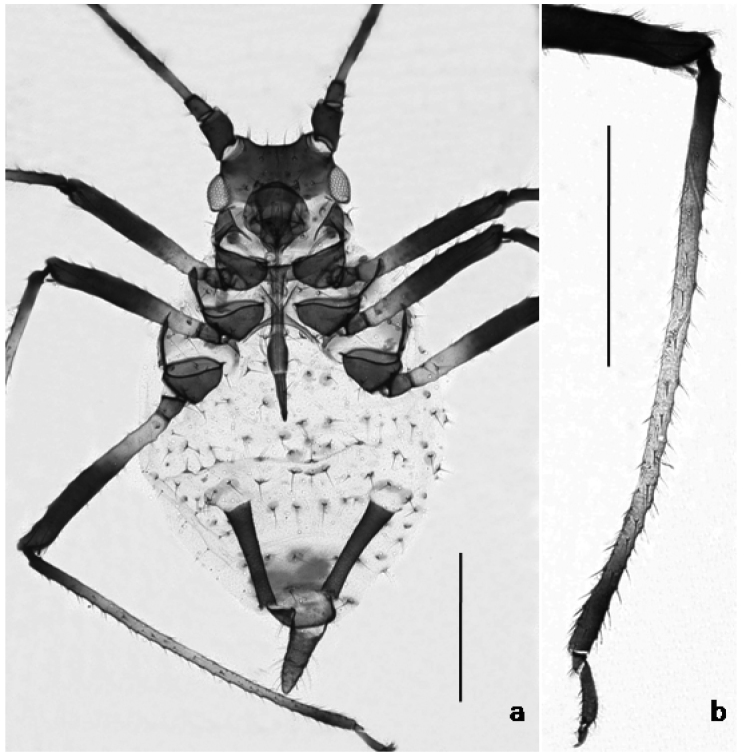
**a** *Macrosiphoniella aetnensis*, apterous viviparous: body shape and pigmentation **b** hind tibia. Scale bars 0.5 mm.

**Figure 3.**
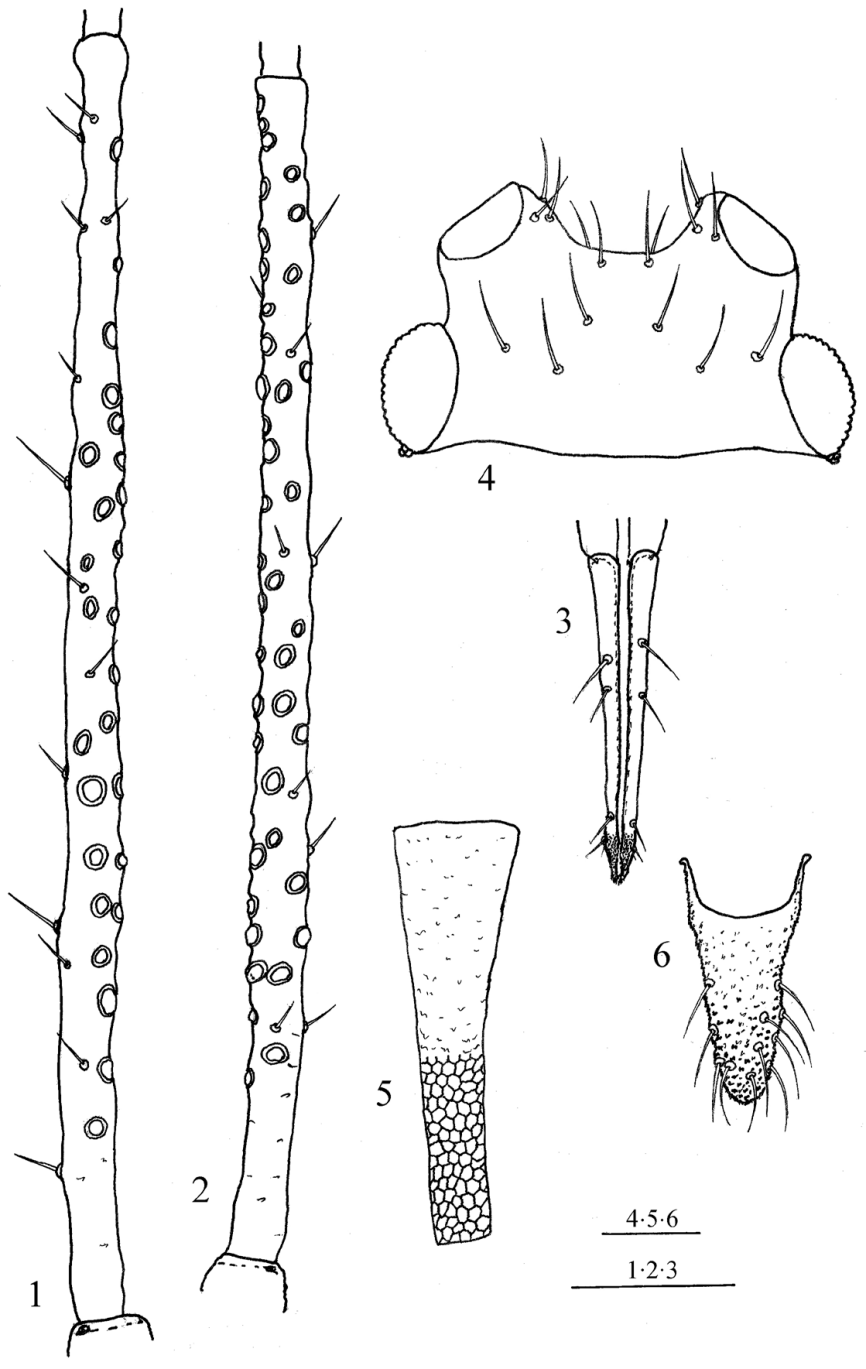
*Macrosiphoniella remaudierei* sp. n. **1** third antennal joint of apterous viviparous **2** same of alate viviparous **3** shape of ultimate rostral joint of apterous viviparous **4** head of apt. viv. **5** siphunculus of apt. viv. female **6** cauda of apt. viv. female. Scale bars 0.1 mm.

**Table 1.** *Macrosiphoniella remaudierei* sp. n. – Apterous (ns. 1–10) and alate (ns. 11–12) viviparous females. Measurements in mm of some type specimens. Abbreviations: Bl, body length; Al, Antennal length; Ajl, Antennal joints length; SrIII, Secondary rhinaria on III ant. joint; Urj, Ultimate rostral joint; IIht, second hind tarsomer; Sl, Siphuncular length; lg, caudal length; hs., number of caudal hairs.

No.	Bl	Al	Ajl				SrIII	Urj	IIht	Sl	Cauda	
			III	IV	V	VI					lg.	hs.
1	2.02	3.18	0.77	0.51	0.47	0.20+1.00	28/27	0.195	0.153	0.45	0.27	12
2	1.96	2.89	0.71	0.52	0.44	0.19+0.82	28/30	0.180	0.137	0.43	0.27	10
3	2.25	3.03	0.74	0.58	0.48	0.19+0.81	37/34	0.188	0.144	0.50	0.28	12
4	2.11	2.75	0.68	0.48	0.44	0.16+0.78	24/25	0.172	0.144	0.45	0.26	10
5	2.15	2.87	0.70	0.51	0.46	0.19+0.80	26/24	0.179	0.146	0.45	0.28	14
6	2.06	2.63	0.65	0.44	0.41	0.16+0.76	26/23	0.181	0.144	0.44	0.26	10
7	2.01	2.66	0.65	0.49	0.41	0.16+0.74	25/24	0.186	0.143	0.44	0.25	14
8	2.07	2.75	0.68	0.52	0.41	0.16+0.78	31/28	0.177	0.141	0.46	0.27	12
9	1.65	2.46	0.60	0.41	0.40	16+0.69	13/17	0.145	0.130	0.37	0.20	10
10	1.76	2.46	0.56	0.44	0.38	0.18+0.71	14/15	0.150	0.132	0.34	0.18	11
11	2.38	3.43	0.84	0.58	0.57	0.20+1.00	38/36	0.188	0.152	0.43	0.30	11
12	2.05	2.79	0.68	0.49	0.44	0.18+0.80	36/40	0.173	0.136	0.36	0.24	9

No. 1 is the **holotype**, others are **paratypes**; ns. 1–3 and 11 sample no. i 515a; ns. 4–8 and 12 sample no. i 834; ns. 9–10 sample no. i 3744. For samples – data see the text (Types).

#### Types.

All type specimens came from three samples collected in Iran from *Helichrysum armenium* DC. by Prof. G. Remaudière. The holotype is an apterous viviparous female (see table 1, specimen no. 1) collected 25 km east of Tehran (35.48 N/51.34 E), 11.VI.1955, 2000 m a.s.l. (sample no. i 515a). Paratypes are 50 apterous and 2 alate viviparous females collected as follows: 1. locality and date as for the holotype, 13 apterae and 1 alate (sample no. i 515a); 2. Dali Tchahi [from the notes of G. Remaudière], near Yaroud (Qazvin province) (36.31 N/50.20 E), 21.VII.1955, 1900 m a.s.l., 32 apterae and 1 alate (sample no. i 834); 3. fifty km NW of Shahr-e-Babak (Kerman province) (30.24 N/55.05 E), 14.IX.1972, 2400 m a.s.l., 5 apterae (sample no. i 3744). All types have been deposited at the Muséum national d’Histoire naturelle, Paris (France).

#### Etymology.

The new taxon is named in honour of the late Prof. Georges Remaudière, a great leader in aphid taxonomy.

#### Ecology and distribution.

The new aphid species, as far as is known, feeds on the aerial parts of *Helichrysum armenium* in Iranian mountainous regions (range of collecting localities: 1900–2400 m a.s.l., co-ordinates as above). Only viviparous morphs (apterae, alatae, and nymphs) were collected, so the aphid life cycle remains unknown. The aphid is probably monoecious and holocyclic, like *Macrosiphoniella aetnensis* (Barbagallo, 1969), as it is unlikely to survive winter as viviparous morphs at such high altitudes.

The host plant has a mainly Eastern Mediterranean-Iranian distribution, ranging from its western boundary (Lebanon, Syria) eastwards to Iran, through Turkey, Georgia, Armenia, Azerbaijan, and Iraq. The aphid, unless it can survive on additional congeneric host plants, is therefore likely to have a similar biogeographic distribution.

### Taxonomic remarks

The new taxon is strictly allied to *Macrosiphoniella aetnensis*, from which it differs by a small number of morphological features and by the pigmentation of the abdomen, legs, and cauda.

The apterous morph of *Macrosiphoniella remaudierei* is characterized by: a. abdomen entirely pale, including the genital plate; b. legs with slightly pigmented coxae and trochanters usually as pale as the basal part of femora; the latter are brown on the distal two-thirds but have a pale subdistal area; tibiae with the pigmented basal part less extensive than the brown distal part; c. cauda much less pigmented than the siphunculi; d. cauda/siphunculi ratio up to 0.7 (range 0.52–0.68); e. antennal joint III with 12–37 (but usually not less than 18) secondary sensoria, which are distributed over 66–93% of its length.

In contrast, the coxae and trochanters in the apterae of *Macrosiphoniella aetnensis* (Fig. 2) are more pigmented. The femora lack a pale subdistal area. Tibiae with their pigmented basal part as long as or longer than the brown distal part. Abdominal tergites usually have small pigmentations (such as rounded spots at the base of dorsal setae, a small ante-siphuncular sclerite, and a narrow bar on the 8^th^ tergite). Genital plate is brownish, and cauda well pigmented (nearly as dark as the siphunculi). Secondary sensoria 7–15, distributed over 49–65% of the length of joint III. Ratio cauda/siphunculi usually more than 0.7 (0.70–0.88).

Alate morphs are more difficult to separate between the two taxa, because the above differences in pigmentation of the body and appendages are much more attenuated. The number of secondary sensoria on antennal joint III are nevertheless 36–40 in two specimens of *Macrosiphoniella remaudierei* (compared to 21–32 in *Macrosiphoniella aetnensis*), and the cauda/siphunculi ratio is 0.67–0.70 (compared to 0.73–0.87 of the other species). *Macrosiphoniella aetnensis* also frequently (48% of 21 specimens) has a few (1–4) secondary rhinaria on antennal joint IV, which are absent in *Macrosiphoniella remaudierei* (this difference, however, may not be reliable due to the paucity of specimens examined for the latter species).

*Macrosiphoniella remaudierei* is not likely to be confused with any other *Macrosiphoniella* species on *Helichrysum*. The four known species of the subgenus *Ramitrichophorus* have very different morphological features, such as a short triangular cauda, a longer ultimate rostral joint, and furcate or ramose setae on the body or appendages (see Hille Ris Lambers 1947; Heie 1995; Blackman and Eastop 2006–2015). *Macrosiphoniella helichrysi* and the very similar *Macrosiphoniella madeirensis* are also quite different, with a dark brown colour in life, extensive pigmentation of the appendages, large dorsal abdominal sclerifications, and other differences (Remaudière 1952; Aguiar and Ilharco 2005). *Macrosiphoniella olgae*, another species sometimes on *Helichrysum* (recorded on *Helichrysum punctatum* by Nevsky 1951), is also brownish in colour but usually feeds on the allied *Gnaphalium* (= *Omalotheca*) *sylvaticum* (Blackman and Eastop 2006–2015; Holman 2009). This rare aphid has only been reported from mountainous regions in Central Asia (Kazakhstan, Uzbekistan) (Nevsky 1929, 1951; Kadyrbekov 2002), even though its main host plant is widespread in western Europe. This aphid is quite different from our new taxon by having (after Nevsky 1929): dark cauda and almost the same length as siphunculi, antennal joint III with 10–20 secondary rhinaria on its basal half, abdomen with small sclerites at the base of the dorsal setae, a well-developed ante-siphuncular sclerite, and other morphological differences.

Among the *Macrosiphoniella* species not found on *Helichrysum* or the allied Inuleae (Asteraceae), *Macrosiphoniella usquertensis* Hille Ris Lambers is widespread in Europe on the *Achillea millefolium* group, and the general aspect of the body pigmentation when mounted on slides roughly resembles that of our new taxon. It differs, however, in the conical-shaped last rostral joint, which is shorter than the second joint of hind tarsus, and in other morphological features (see Hille Ris Lambers 1938, Heie 1995).

## Supplementary Material

XML Treatment for Macrosiphoniella remaudierei
